# Investigating the hydrolysis-condensation of dichloromethylsilane: a simplified protocol for polymethylhydrosiloxane synthesis as a candidate for vitreous substitute

**DOI:** 10.1186/s13065-026-01862-6

**Published:** 2026-06-15

**Authors:** Mutiara Septiani, Diba Grace Auliya, Lukluk Marfu’ah, Lena Juanda, Lusi Safriani, Wan Nurfadhilah Zaharim, Atiek Rostika Noviyanti, Risdiana Risdiana

**Affiliations:** 1 Department of Chemistry, Faculty of Mathematics and Natural Sciences, Universitas Padjadjaran, Jl. Raya Bandung-Sumedang Km. 21, Jatinangor, Sumedang, 45363 Indonesia; 2 Department of Physics, Faculty of Mathematics and Natural Sciences, Universitas Padjadjaran, Jl. Raya Bandung-Sumedang Km. 21, Jatinangor, Sumedang, 45363 Indonesia; 3 Department of Chemical Sciences, Faculty of Science and Technology, Universiti Kebangsaan Malaysia, 43600 Bangi, Selangor Malaysia

**Keywords:** Dichloromethylsilane, Polymethylhydrosiloxane, Hydrolysis–condensation, Vitreous substitute, Viscosity

## Abstract

This study reports a facile and efficient synthesis of polymethylhydrosiloxane (PMHS) via a simplified hydrolysis–condensation of dichloromethylsilane (DCMS), eliminating the need for a separate multistep condensation stage. PMHS samples were prepared by controlling reaction time and temperature and subsequently characterized for viscosity, refractive index, surface tension, and optical transmittance. Viscosity values were tuned from low (1.01 Pa·s) to medium (2.00 Pa·s), and the maximum isolated yield reached 34%, improving upon the previously reported 29.8% yield. All samples exhibited refractive indices between 1.3962 and 1.3980 and additional diopters in the range +3.02 D to +3.12 D, consistent with clinical requirements for vitreous substitutes. FTIR and ^1^H‑NMR analyses confirmed the presence of Si–H groups and the Si–O–Si backbone, indicating preservation of key functional groups during synthesis. The simplified hydrolysis–condensation protocol provides a simpler, faster route to PMHS with tunable properties suitable for further biomedical evaluation.

**Graphical Abstract:**

**Figure:**
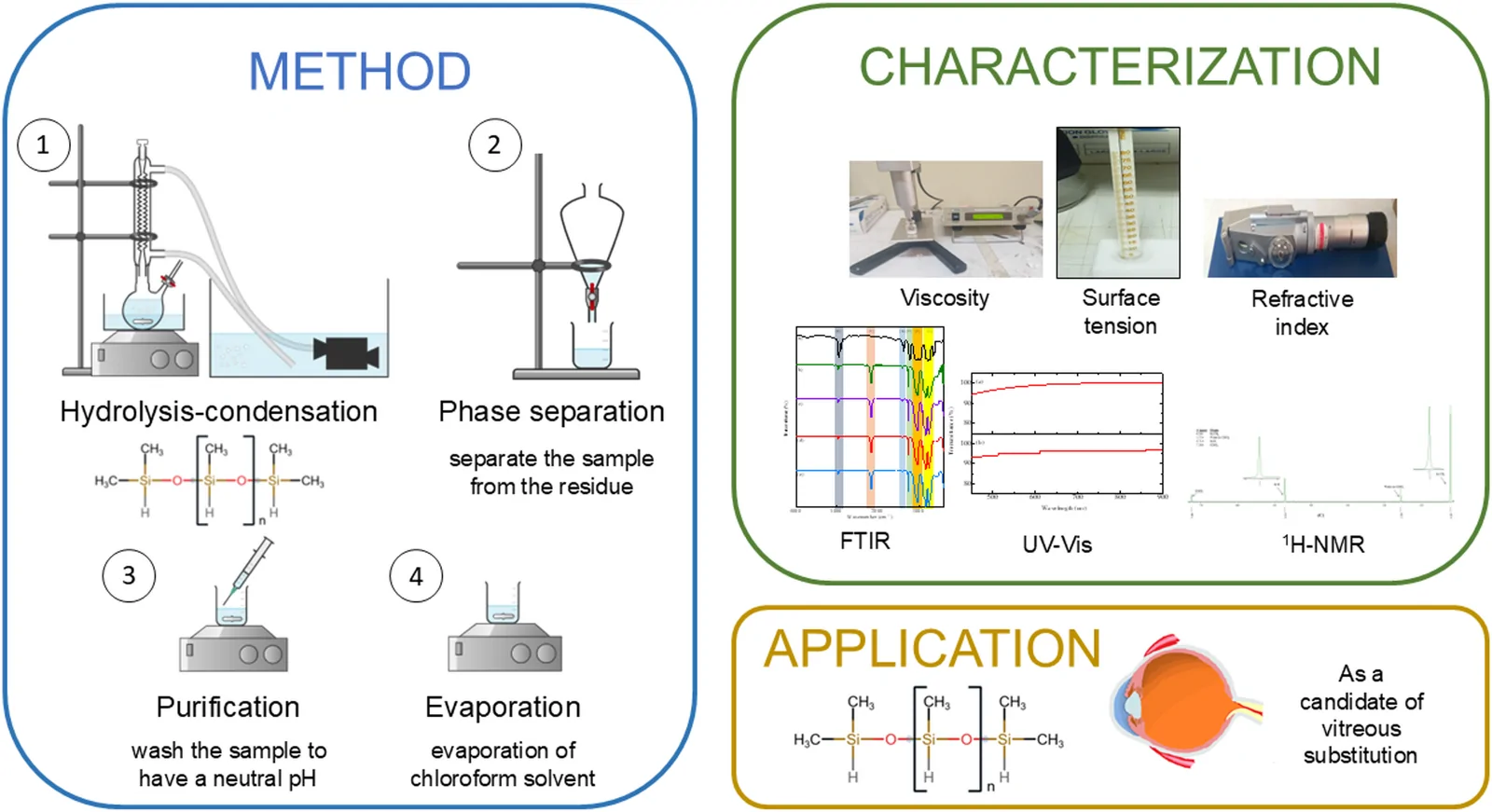

## Introduction

The vitreous humor is a transparent gel that maintains ocular shape and protects internal structures from friction and vibration. The vitreous humor can shrink or become damaged, causing the neurosensory retinal segment to separate from the retinal epithelium. This separation is also known as retinal detachment, which can then lead to blindness [1–5]. This condition requires vitreoretinal surgery to replace the damaged vitreous humor with a suitable vitreous substitute. Polydimethylsiloxane (PDMS) has become the most commonly used material for this purpose. However, PDMS synthesis, typically via ring-opening polymerisation (ROP) of octamethylcyclotetrasiloxane (D4) monomers, faces challenges due to the difficulty in accessing the D4 monomer [6, 7].

To overcome these limitations, research has shifted towards developing alternative materials, with polymethylhydrosiloxane (PMHS) emerging as a highly promising candidate. PMHS is known to be transparent, stable, and non-toxic, making it suitable for biomedical applications [8–10]. Commercial PMHS typically exhibits very low viscosity (≈ 15–40 mPa·s), which is insufficient for vitreous replacement where viscosities comparable to commercial PDMS (≥ 0.95 Pa·s and up to ≈ 5.5 Pa·s) are required [11–14]. Therefore, the development of an efficient synthesis method to produce PMHS with a viscosity matching commercial PDMS is necessary.

Arini et al. (2022) successfully synthesised PMHS from dichloromethylsilane (DCMS) precursor using a hydrolysis-condensation method for 3 h to obtain short chains of PMHS, followed by an additional condensation process to form long chains over 72 days [15]. Nurmadanti et al. (2024) controlled the synthesis temperature and used potassium hydroxide (KOH) as a catalyst, reducing the condensation time from 72 days to just 2 h to produce long-chain PMHS [6]. However, behind this progress, two major challenges remain. First, the resulting product still falls within the low viscosity group (0.93–1.10 Pa·s) with a relatively low yield (~29.8%) [6, 7]. From a clinical perspective, this low-viscosity profile poses a significant limitation for vitreous substitutes, as low-viscosity siloxanes are highly susceptible to intraocular emulsification over time, potentially leading to severe complications such as secondary glaucoma [16, 17]. Second, these methods essentially rely on a distinct two-step process: an initial hydrolysis-condensation step to form short-chain polymers, followed by a separate condensation step to produce long-chain PMHS. This multi-step approach, while effective, opens up opportunities for further simplification to create a more concise and efficient production route by focusing on the results of the physical properties of PMHS to match the physical properties of the material as a substitute for vitreous humor.

This study aims to develop a simplified hydrolysis–condensation method that eliminates the need for a separate condensation step. By systematically varying reaction time and temperature and optimizing solvent ratios, we target PMHS with tunable viscosity in the clinically relevant range and improved yields. We benchmark the resulting materials against commercial PDMS for viscosity, refractive index, surface tension, transmittance, and chemical structure [18, 19].

## Materials and methods

PMHS was synthesized using the hydrolysis-condensation method, with temperature and reaction time as key parameters. The chemical reaction for the synthesis of PMHS by this method is shown in Eq. (1).

1 $$\begin{aligned} & {\mathrm{nCH}}_{3} {\mathrm{SiHCl}}_{2} + 2{\mathrm{n}}~{\mathrm{H}}_{2} {\mathrm{O}} \to {\mathrm{n}}~{\mathrm{CH}}_{3} {\mathrm{SiH}}\left( {{\mathrm{OH}}} \right)_{2} + 2{\mathrm{n}}{\mathrm{HCl}} \hfill \\& {\mathrm{nCH}}_{3} {\mathrm{SiH}}\left( {{\mathrm{OH}}} \right)_{2}\! +\! {\mathrm{CH}}_{3} {\mathrm{SiH}}\left( {{\mathrm{OH}}} \right)_{2} \to {\mathrm{OH}}\! -\! \left( { - {\mathrm{CH}}_{3} \left( {\mathrm{H}} \right){\mathrm{SiO}} \!-\! } \right)_{{{\mathrm{n}} + 1}}\! -\! {\mathrm{H}}\! +\! {\mathrm{n}}{\mathrm{H}}_{2} {\mathrm{O}} \hfill \\ \end{aligned} $$

As illustrated by the chemical reaction in Eq. (1), the materials used in this synthesis were dichloromethylsilane (DCMS, 97%) as a precursor obtained from Sigma-Aldrich, dichloromethane (DCM, 99.8%) as a solvent, purchased from Merck, and milli-Q water. The synthesis was carried out in a heterogeneous biphasic system (aqueous/organic) due to the immiscibility of DCM and water. Given the low boiling points of both DCM and DCMS (approximately 40 °C) and the exothermic nature of the hydrolysis reaction, the reaction flask was equipped with a reflux condenser to prevent the volatilization and loss of reactants. The reaction was initiated by mixing 25 mL of DCMS, 25 mL of DCM, and 12.5 mL of Milli-Q water (a volume ratio of 2:2:1) [6, 20]. The synthesis was systematically conducted under several temperature and time parameters: 35 °C (for 35, 45, 50, 55, 60, 100, and 125 min), 40 °C (for 35, 50, and 125 min), and 45 °C (for 25 and 35 min).

Upon completion of the specified reaction time, stirring was ceased, and a distinct phase separation was visually observed. The organic phase, containing the synthesized PMHS, was subsequently separated from the aqueous layer. The product was then purified using chloroform and repeatedly washed with Milli-Q water until the residual acid was completely removed and a neutral pH was achieved [6, 7].

All physical and chemical characterizations were conducted at a controlled room temperature of 20 °C. The properties were evaluated using a Sekonic Viscomate VM-10 A-MH digital viscometer for viscosity, and the refractive index was determined using an AS ONE I-500 handheld refractometer (Brix 0–90%). Surface tension was precisely measured using a DG-1 capillary-rise surfgauge. A minimal volume of the synthesized PMHS sample was dispensed into a Teflon dish, and the capillary tube was vertically immersed. Upon reaching hydrostatic equilibrium, the surface tension value was directly recorded from the integrated graduated scale (0–80 mN/m) with a resolution of 1 mN/m. Optical transmittance was measured using a PG Instruments T + 70 UV-Vis spectrometer equipped with a standard quartz cuvette. Functional-group analysis was performed on a PerkinElmer Spectrum 100 FT-IR spectrometer, recording the spectra in the wavenumber range of 4000–400 cm^− 1^. Molecular characterization was conducted using a 500 MHz Agilent VNMRS500 spectrometer; the ¹H-NMR spectra were recorded in CDCl_3_, using the residual solvent peak as a reference. The additional diopters were determined by Eq. (2).

2 $$\:\left( {\frac{{N_{S} - N_{v} }}{{AL - ACD}}} \right) \times \:100 = {\text{Additional diopters}}$$

where N_s_ is the refractive index value of PDMS, N_v_ is the refractive index value of vitreous humor (1.3348), AL is axial length (23.35 mm), and ACD is anterior chamber depth (3.06 mm) [21].

## Results and discussion

The synthesis of polysiloxanes, such as PMHS, is a fundamental process in polymer chemistry that relies on the hydrolysis-condensation method. This is a reaction that involves the controlled reaction of an organosilane precursor with water. Hydrolysis occurs when a molecule of water attacks the silicon atom of the precursor (CH_3_HSiCl_2_), cleaving off a reactive group like chlorine (-Cl) or alkoxy (-OR) and replacing it with a hydroxyl group (-OH). This reaction is very fast and forms an unstable intermediate known as a silanol (CH_3_HSi(OH)_2_) [22–24]. Condensation occurs when two silanol intermediates react with each other. This reaction results in the formation of a stable siloxane bond (-Si-O-Si-) while a small molecule, typically water (H_2_O), is eliminated. This process repeats continuously, forming long polymer chains of PMHS. The balance between hydrolysis and condensation is critical for controlling the final structure of the polymer, as these two reactions compete with each other [25].

Several parameters must be carefully controlled to achieve the desired properties of the final PMHS polymer. The precursor-to-water ratio is one of the most important factors. Excess water can accelerate hydrolysis, leading to rapid condensation and potentially forming a cross-linked gel instead of a linear polymer. Conversely, insufficient water can result in incomplete hydrolysis and the formation of low-molecular-weight oligomers. The selection of the volume ratio of Milli-Q water to DCMS precursor was based on previous research that successfully obtained PMHS with the desired physical characteristics [6, 7].

The type and concentration of the catalyst, either acid or base, also significantly affect the reaction. Acid primarily catalyzes the hydrolysis step, while base is very effective in accelerating the condensation of silanol groups. An appropriate catalyst can significantly increase the reaction rate and influence the molecular weight distribution of the polymer. The hydrolysis-condensation reaction of PMHS produces an acidic waste product that is then utilized in the synthesis process of this study, making it possible to obtain long-chain PMHS without further condensation [7, 24]. This continuous chain growth is facilitated by the immiscibility of the biphasic DCM/water system [26, 27]. Because the organic DCM layer inherently dissolves a very limited amount of water, it creates a favorable environment for the silanol intermediates. Consequently, the generated acid can catalyze the polycondensation reaction without being hindered by the overall excess of water [22].

Reaction temperature and time are other important factors. Higher temperatures generally increase reaction rates, but careful temperature control is essential to prevent uncontrolled reactions that can lead to gel formation. The reaction time is proportional to the viscosity of the PMHS. The longer the hydrolysis-condensation reaction time, the higher the viscosity obtained [28–32]. However, the synthesis time also requires careful consideration, since prolonged reaction time can promote gel formation and loss of polymer flowability. In addition, the type of solvent used can also affect the reaction by dissolving the reactants and moderating the reaction rate and temperature [7, 33].

In this study, PMHS was synthesized using a hydrolysis-condensation method without a further condensation step. This approach is based on the theoretical assumption that by varying parameters such as temperature and time, a polymer with physical characteristics comparable to commercial PDMS can be produced. To validate this, a direct benchmark comparison was performed. The key physical characteristics of the synthesized PMHS samples, alongside the clinical benchmark (commercial PDMS), are detailed in Table 1.

**Table 1:** Characteristic results of PMHS and commercial PDMS

Sample	T(°C)	t (minutes)	η (Pa s)	γ (mN/m)	*n*	+diopters	Yield (%)
S-1	35	35	0.15	17	1.3973	3.08	36
S-2		45	0.63	19	1.3962	3.03	36
S-3		60	0.71	20	1.3966	3.04	36
S-4		100	1.01	19	1.3972	3.02	34
S-5		125	1.21	21	1.3980	3.12	32
S-6	40	35	0.71	20	1.3962	3.05	34
S-7		50	1.15	21	1.3968	3.05	32
S-8		125	2.00	20	1.3980	3.12	34
S-9	45	25	0.92	21	1.3974	3.09	32
S-10		35	1.22	21	1.3962	3.03	30
S-11		45	1.44	20	1.3964	3.04	30
Low viscosity Commercial [19]	–	–	1.08	20	1.4044	3.43	–

The synthesis results in this study confirm that long-chain PMHS can be successfully prepared using a simplified method, eliminating the need for a separate condensation process. This method presents a notable improvement in efficiency. For comparison, previous research by Arini et al. required a 3-h hydrolysis step followed by an additional condensation process of 72 days to achieve low viscosity PMHS [15]. Meanwhile, Nurmadanti et al. produced low viscosity PMHS with an additional condensation process for 2 h at a temperature of 50 °C [6]. Furthermore, both require substantially larger solvent volumes, threefold [6] and fourfold [15], than those used in our method.

**Fig. 1:**
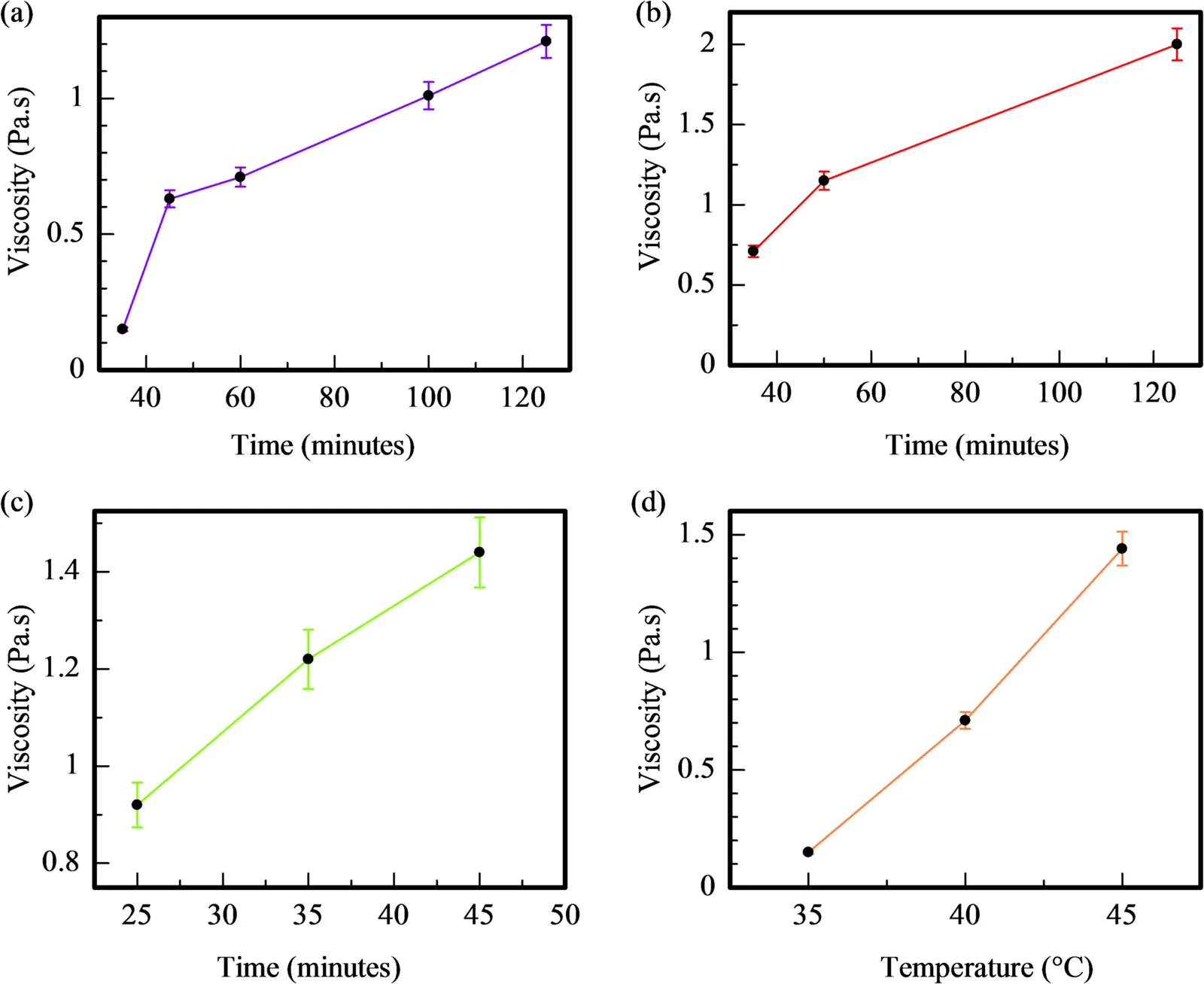
Samples viscosity as a function of time **a** 35 °C, **b** 40 °C, **c** 45 °C, and temperature (**d**) 35 min

This achievement is demonstrated by the viscosity variation obtained under various reaction conditions. According to Table 1, the highest viscosity was achieved by sample S-8, reaching 2 Pa·s. This value places S-8 in the medium viscosity category, approaching the viscosity range of medium viscosity commercial PDMS (2.00–2.40 Pa·s) [18]. Meanwhile, samples S-4, S-5, S-7, S-9, S-10, and S-11 were categorized as low viscosity, with values comparable to the commercial range of 0.90–1.80 Pa·s [18]. In the context of vitreous humor substitutes, reaching this medium-viscosity range is highly preferable over the low-viscosity variant. Previous clinical and biomaterial studies have demonstrated that low-viscosity siloxanes are highly prone to intraocular emulsification over time, which can lead to severe complications. By yielding a medium-viscosity polymer, the material possesses better rheological resistance against dispersion. This higher viscosity helps mitigate the risk of emulsification while ensuring the polymer remains optimal for intraocular applications [16–18]. The effect of variations in synthesis parameters on the viscosity of PMHS is shown in Fig. 1.

As illustrated in Fig. 1a–c, viscosity tends to increase with longer reaction duration due to the remaining active silanol (-OH) groups continuing to react to promote further polymer chain elongation. Apart from reaction time, temperature also proves to be a crucial factor [34, 35]. Figure 1d shows that increasing synthesis temperature is positively correlated with increased viscosity. Higher thermal energy facilitates the polymerization reaction by providing sufficient energy to overcome the activation energy threshold. This accelerates the rate of siloxane (Si-O-Si) bond formation, affects the molecular weight distribution, and ultimately produces polymers with longer chains and higher viscosity [35].

The surface tension (γ) values for all synthesized PMHS samples were found to be within the range of 19 mN/m to 21 mN/m for the samples with viscosities corresponding to viscosity group of commercial PDMS. This range corresponds to the standard commercial PDMS value, which is measured at 20 mN/m [19]. This surface tension value is crucial for applications of materials intended as vitreous replacements, as it significantly impacts the performance and compatibility of the sample. This tension is essential for effectively sealing retinal tears and preventing fluid migration into the subretinal space, which is a key mechanism for retinal reattachment [18]. Higher surface tension values improve the stability and quality of the vitreous substitute [36].

The synthesized PMHS samples exhibited refractive indices ranging from 1.3962 to 1.3980. These values fall within the acceptable range of optical properties for the intended application [21]. The calculated additional diopters for all samples were within the clinically acceptable range for vitreous humor substitutes (+3.0D to +3.5D). The additional diopter value obtained is lower than low commercial PDMS, which is +3.43D [19, 21].

The yields for the synthesized samples range from 30% to 36%. The efficiency of the synthesis process was evaluated by calculating both the product yield and the synthesis time required to obtain the desired properties. Although a maximum isolated yield of 36% was recorded under certain conditions, that sample did not meet the viscosity criterion for a vitreous substitute; therefore, the highest yield among samples that satisfied the target viscosity was 34% (S‑4 and S‑8). This improvement relative to previously reported values (~29.8%) is attributed primarily to the simplified protocol, which reduces material loss from inter‑step transfers and lowers solvent consumption [6].

**Fig. 2:**
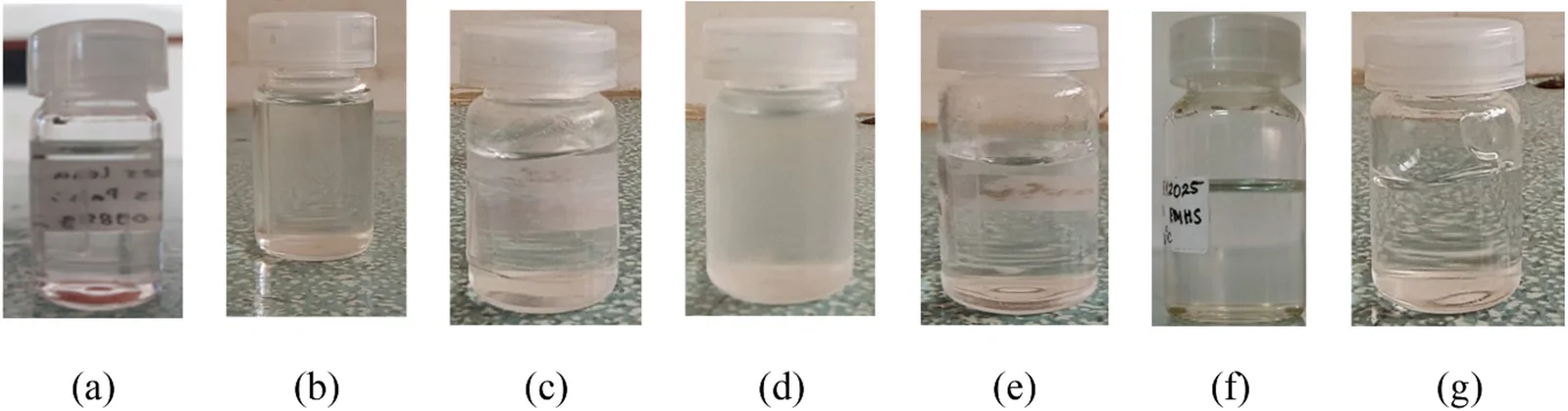
Physical appearance PMHS samples of **a** S-4, **b **S-5, **c** S-7, **d** S-8, **e** S-9, **f** S-10, and **g** S-11

**Fig. 3:**
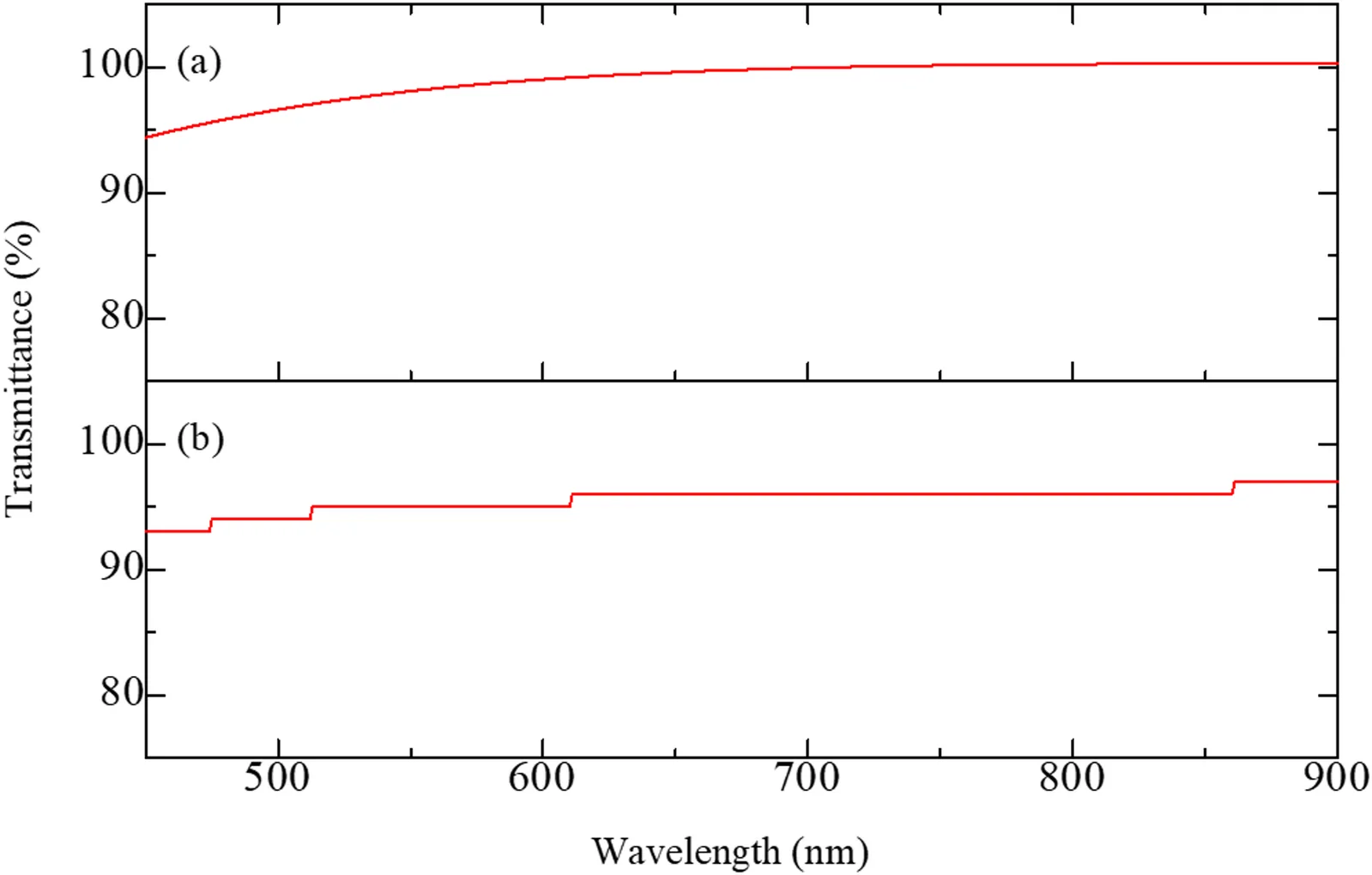
Transmittance in the visible wavelength range of **a** S-4 and **b** S-8

Figure 2 illustrates the physical appearance of the PMHS samples synthesized at different temperatures. A clear, transparent liquid was obtained for most samples, specifically S-4 (Fig. 2a), S-7 (2c), S-9 (2e), S-10 (2f), and S-11 (2g). However, sample S-8 (Fig. 2d) appeared slightly opaque. To quantitatively evaluate these optical properties, UV-Vis transmittance spectra were recorded and are shown in Fig. 3. The data strongly support the visual assessments. The clear sample of S-4 demonstrated high optical transparency, with transmittance values ranging from 91 to 100% across the visible spectrum (Fig. 3a). In contrast, the slightly opaque appearance of S-8 was correlated with a lower optical transmittance, which ranged from 84 to 97% (Fig. 3b), confirming its reduced clarity compared to S-4. The high S-4 transmittance indicates that the sample is optically homogeneous, meaning its structure is uniform at the microscopic level. This allows light to pass through it directly with minimal interference. In contrast, the lower S-8 transmittance is due to light scattering. As previously discussed, the extended reaction time (125 min) under acidic in situ conditions likely triggered an uncontrolled polymerization rate. Theoretically, such conditions are expected to lead to a broader molecular weight distribution (higher PDI) [35]. The suspected coexistence of polymer chains with significantly different lengths is likely responsible for creating the internal structural inhomogeneities [37]. When light waves hit these features, they are bent from their straight path into different directions, which reduces the clarity of the material and reduces the total amount of light that passes through it directly [38, 39].

**Fig. 4:**
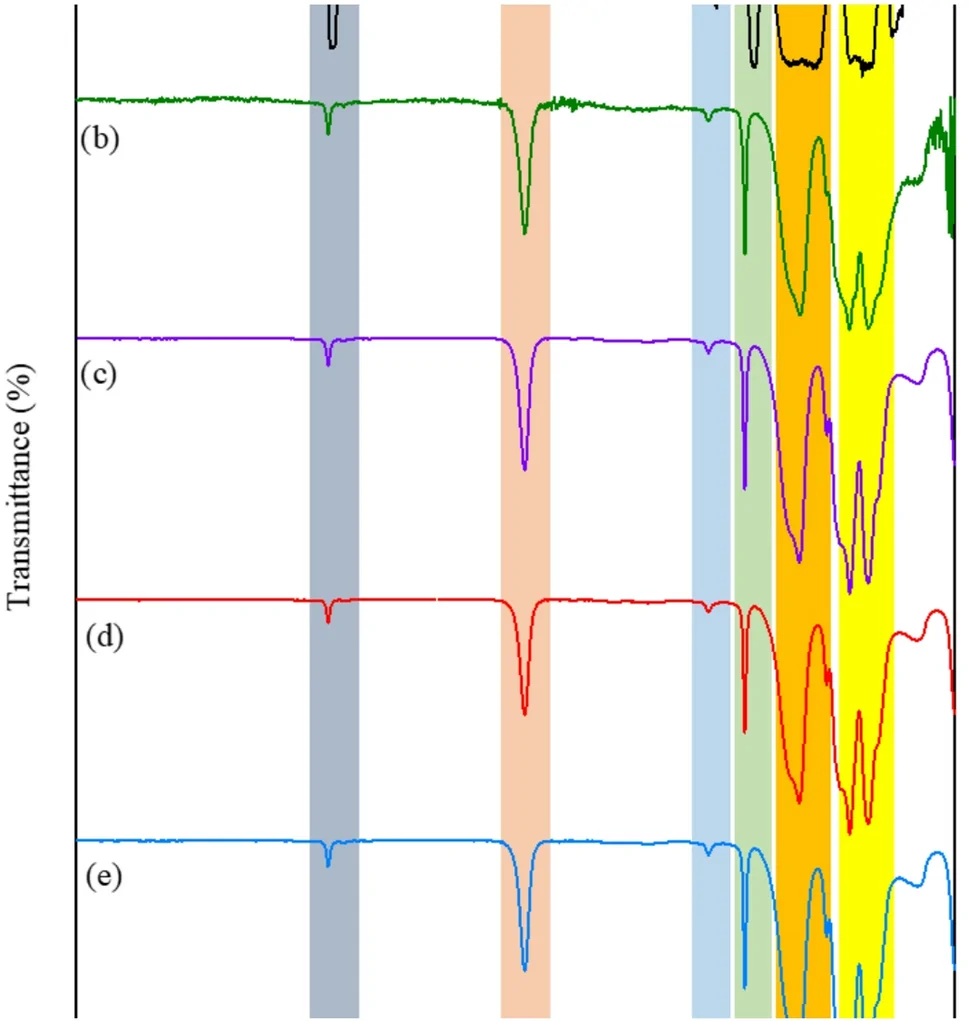
IR spectra of **a** low viscosity commercial PDMS [19], **b** literature PMHS [7], **c** S-9, **d** S-4, and **e** S-8

Figure 4 shows the IR spectra of commercial PDMS, literature PMHS, and the synthesized PMHS samples of S-9, S-4, and S-8. The functional groups that have been identified from the IR spectra are listed in Table 2. The FTIR spectra of samples S-4 exhibit the characteristic absorption patterns of siloxane compounds. In Table 2, a prominent absorption peak is observed at approximately 1035.7 cm⁻¹, corresponding to the stretching vibration of the Si–O–Si bond. The presence of this strong peak, which is characteristic of the siloxane backbone, confirms the successful formation of the polymer chain via the condensation of silanol groups. This wavenumber closely matches the literature value for PMHS (1032.37 cm⁻¹) and shows a slight shift compared to commercial PDMS (1022.8 and 1111.9 cm⁻¹), which can be attributed to the structural differences between the two polymers [6, 7, 40, 41].

A key distinguishing feature of PMHS is the presence of the Si–H functional group, which is absent in PDMS. This group is identified by a characteristic absorption peak at 2161.2 cm⁻¹. The appearance of this distinct peak, which is not found in the spectrum of commercial PDMS, unequivocally confirms that the synthesised product is PMHS. Furthermore, this peak’s position is consistent with the reported literature data for PMHS (2158.92 cm⁻¹), indicating that the Si–H functional groups were successfully preserved throughout the hydrolysis-condensation process [7, 15, 42, 43]. Collectively, the IR spectrum of the synthesised product (e.g., sample S-4) demonstrates a high degree of similarity to the reference spectrum of PMHS from the literature, regarding both peak positions and the types of functional groups identified.

**Table 2:** Functional groups of commercial PDMS [19], literature PMHS [7], S-9, S-4, and S-8

No	Functional Groups	Wavenumber (cm^− 1^)				
		Commercial PDMS [19]	Literature PMHS [7]	S-9	S-4	S-8
(1)	Si-C stretching and CH_3_ rocking	792–823.8	753.69–828.69	753.4–829.1	753.6–828.9	753.4–828.6
(2)	Si-O-Si stretching	1022.8; 1111.9	1032.37	1035.7	1035.88	1035.32
(3)	CH_3_ symmetric deformation of Si-CH_3_	1263	1258.66	1259.2	1259.29	1259.23
(4)	CH_3_ asymmetric deformation of Si-CH_3_	1412.3	1405.10	1408.1	1408.07	1408.14
(5)	Si-H	–	2158.92	2161.3	2161.2	2160,99
(6)	CH stretching of CH_3_	2905.5; 2971.7	2965.71	2966.5	2966.40	2966.37

**Fig. 5:**
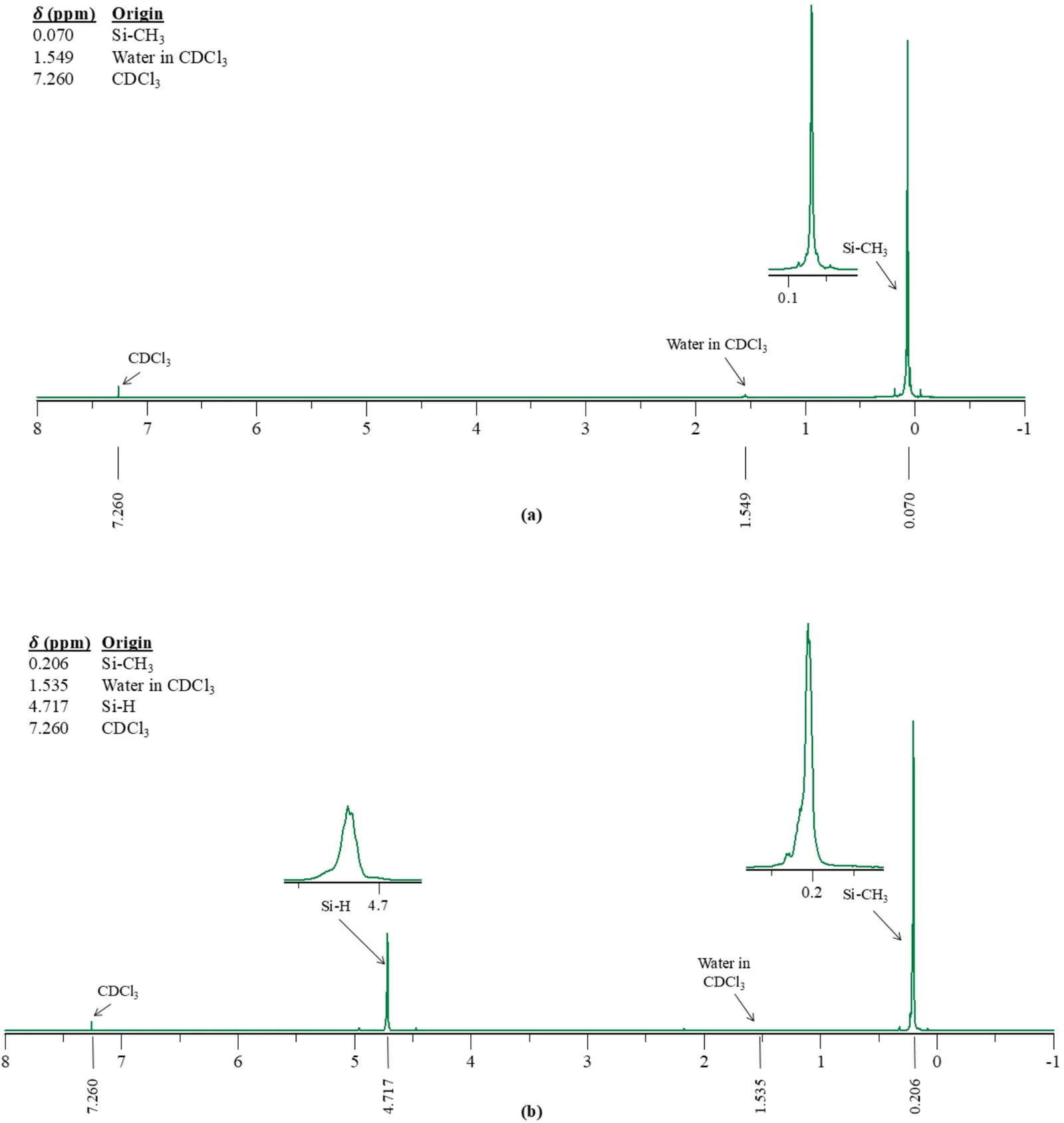
¹H-NMR spectra of commercial PDMS (**a**) [44], literature PMHS (**b**) [6], and S-4 sample (**c**)

To further elucidate the chemical structure and confirm the successful synthesis of PMHS, ¹H-NMR spectroscopy was performed. Figure 5 presents the ¹H-NMR spectra of commercial PDMS (Fig. 5a), literature PMHS (Fig. 5b), and the synthesized PMHS sample S-4 (Fig. 5c). In the spectrum of commercial PDMS, a single dominant peak is observed at $\:\delta\:$ = 0.070 ppm, which corresponds to the protons of the dimethylsiloxane repeating units (Si–CH₃). Crucially, the absence of a signal near 4.7 ppm confirms the lack of Si–H bonds in the PDMS structure [44].

In contrast, both the literature PMHS and the synthesized S-4 sample exhibit two characteristic primary peaks. The prominent signal at $\:\delta\:$= 4.714 ppm in sample S-4 (analogous to 4.717 ppm in the literature reference) is distinctly attributed to the proton directly attached to the silicon atom (Si–H). Additionally, the intense peak at $\:\delta\:$= 0.201 ppm (0.206 ppm in the literature) corresponds to the protons of the methyl group attached to the silicon atom (Si–CH₃) [6, 45]. Minor resonance peaks were observed at approximately $\:\delta\:$= 7.260 ppm and $\:\delta\:$= 1.55 ppm in all spectra are attributed to the residual non-deuterated chloroform solvent CDCl_3_ and trace moisture impurities, respectively. The unambiguous presence of the Si–H peak in sample S-4, perfectly mirroring the literature reference, strongly confirms that the polymethylhydrosiloxane backbone was successfully formed [45, 46]. This confirms the preservation of the Si–H functional groups in the synthesized polymer. In the present work, the successful formation of the targeted PMHS structure is comprehensively confirmed by the distinctive characteristic peaks observed in both the FT-IR and ^1^H-NMR profiles, which align strongly with established literature [6, 7]. Additionally, the measured viscosity serves as an indirect indication of the polymer chain information and elongation. For future investigations, the integration of ^29^Si-NMR spectroscopy combined with Gel Permeation Chromatography (GPC) is highly recommended to definitively elucidate the macro-structural configurations, such as distinguishing between linear and cyclic polysiloxane structures, and comprehensively analyze the molecular weight distribution of this chemical material.

The hydrolysis-condensation method, applied without further condensation, proved to be an effective strategy for the synthesis of PMHS. This process successfully controlled the polymer viscosity, produced physicochemical properties comparable to commercial standards of vitreous humor substitute material. Notably, this method achieved a yield of 34% and maintained a consistent chemical structure as validated by FTIR and ^1^H-NMR.

## Conclusion

In this study, PMHS was successfully synthesized via a simplified hydrolysis–condensation method that eliminates the need for a secondary condensation step. Reaction time and temperature were identified as critical parameters for controlling the final polymer properties, particularly viscosity (1.01–2.00 Pa·s), with yields ranging from 30% to 34%. All synthesized samples exhibited physical and optical properties (surface tension, refractive index, and additional diopters) that are comparable to commercial PDMS and meet the requirements for potential vitreous substitute candidates. FTIR and ^1^H-NMR analyses consistently confirmed the desired PMHS chemical structure, explicitly verifying formation of the siloxane (Si–O–Si) backbone and preservation of the Si–H functional groups across the analyzed samples. Collectively, these results indicate that the simplified hydrolysis–condensation route is a practical and efficient approach for producing tailored PMHS as a candidate for vitreous substitute. Future investigations will incorporate ^29^Si-NMR, Gel Permeation Chromatography (GPC), and comprehensive toxicity testing to strengthen these findings and evaluate the biocompatibility of the synthesized polymer for vitreous substitution.

## Data Availability

All data generated or analysed during this study are included in this published article.

## References

[1] Mishra D, Gade S, Glover K, Sheshala R, Singh TRR. Vitreous humor: composition, characteristics and implication on intravitreal drug delivery. Curr Eye Res. 2023. 10.1080/02713683.2022.2119254. 36036478 10.1080/02713683.2022.2119254

[2] Monteiro JP, Santos FM, Rocha AS, Castro-de-Sousa JP, Queiroz JA, Passarinha LA, et al. Vitreous humor in the pathologic scope: insights from proteomic approaches. Proteomics Clin Appl. 2015;9(1–2):187–202. 25523418 10.1002/prca.201400133

[3] Khurana AK. Comprehensive Ophthalmology. 4th ed. New Delhi: New Age International; 2007.

[4] Peng Y, Yu Y, Lin L, Liu X, Zhang X, Wang P, et al. Glycosaminoglycans from Bovine Eye Vitreous Humour and Interaction with Collagen Type II. Glycoconj J. 2018;35(1):119–28. 29305777 10.1007/s10719-017-9808-1 PMC5851830

[5] Tram NK, Swindle-Reilly KE. Rheological Properties and Age-Related Changes of the Human Vitreous Humor. Front Bioeng Biotechnol. 2018;6:199. 30619846 10.3389/fbioe.2018.00199 PMC6305337

[6] Nurmadanti T, Auliya DG, Zahra NF, Mardina SAI, Safriani L, Risdiana R. Synthesis and characterization of polymethylhydrosiloxane using dichloromethane as a candidate material for vitreous substitution. J Polym Res. 2024;31:292.

[7] Auliya DG, Arini VF, Nurmadanti T, Fauziah U, Setiadji S, Fitrilawati F, et al. Tailoring polymethylhydrosiloxane as candidate material for vitreous humour substitution: physical properties and in vitro toxicity. BMC Chem. 2025. 10.1186/s13065-025-01548-5. 40646567 10.1186/s13065-025-01548-5 PMC12255143

[8] Yadav MS, Singh AS, Agrahari AK, Mishra N, Tiwari VK. Silicon industry waste polymethylhydrosiloxane-mediated benzotriazole ring cleavage: a practical and green synthesis of diverse benzothiazoles. ACS Omega. 2019. 10.1021/acsomega.9b00343. 31459794 10.1021/acsomega.9b00343 PMC6648665

[9] Chruściel J, Fejdyś M, Michalska Z, Fortuniak W. Synthesis and characterization of new liquid, branched and random poly(methylhydrosiloxanes). e-Polymers. 2008. 10.1515/epoly.2008.8.1.621. 10.3390/polym10050484 PMC6415389 30966518

[10] Chruściel JJ, Fejdyś M, Fortuniak W, Synthesis. Characterization and microstructure of new liquid poly(methylhydrosiloxanes) containing branching units SiO4/2. Polymers. 2018;10(5):484. 30966518 10.3390/polym10050484 PMC6415389

[11] Donati S, Caprani SM, Airaghi G, Vinciguerra R, Bartalena L, Testa F, et al. Vitreous substitutes: the present and the future. Biomed Res Int. 2014;2014(1):351804, 351804. 24877085 10.1155/2014/351804 PMC4024399

[12] Fitrilawati, Fauza AN, Ardi A, Novianti RM, Syakir N, Kartasasmita AS, et al. Effect of KOH concentration on characteristics of polydimethylsiloxane synthesized by ring opening polymerization method. J Phys Conf Ser. 2018;1080(1):01216.

[13] KGaA M. Sigma Aldrich-Poly(methylhydrosiloxane). Merck KGaA. 2021. https://www.sigmaaldrich.com/catalog/substance/polymethylhydrosiloxane123456314857211?lang=en&region=ID. Accessed 20 Jan 2021.

[14] Auliya DG, Setiadji S, Agasa ZM, Fitrilawati, Syakir N. Risdiana. Synthesis of Low Viscosity Polydimethylsiloxane Using Low Grade of Octamethylcyclotetrasiloxane. Mater Sci Forum. 2021;1028 MSF:365–70.

[15] Arini VF, Fauziah U, Auliya DG, Setiadji S. Synthesis of low viscosity of polymethylhydrosiloxane using monomer of dichloromethylsilane. J Phys Conf Ser. 2022. 10.1088/1742-6596/2165/1/012041.

[16] Branisteanu DC, Moraru AD, Maranduca MA, Branisteanu DE, Stoleriu G, Branisteanu CI, et al. Intraocular pressure changes during and after silicone oil endotamponade (review). Exp Ther Med. 2020;20(6):204. 33123233 10.3892/etm.2020.9334 PMC7588780

[17] Łątkowska M, Gajdzis M, Kaczmarek R. Emulsification of silicone oils: altering factors and possible complications—a narrative review. Journal of Clinical Medicine. 2024. 10.3390/jcm13082407. 38673681 10.3390/jcm13082407 PMC11051299

[18] Caramoy A, Hagedorn N, Fauser S, Kugler W, Groß T, Kirchhof B. Development of emulsification-resistant silicone oils: can we go beyond 2000 mPas silicone oil? Investigative Opthalmology & Visual Science. 2000;52(8):5432–6. 10.1167/iovs.11-7250 21540478

[19] Setiadji S, Fitrilawati, Fauza AN, Ardi A, Novianti RM, Syakir N, et al. Optimization of Polydimethylsiloxane Synthesized Parameters as Vitreous Humour Substitutes. Mater Sci Forum. 2019;966 MSF:189–93.

[20] Auliya DG, Fauziah U, Arini VF, Setiadji S, Fitrilawati F, Kartasasmita AS, et al. Use of dichlorodimethylsilane to produce polydimethylsiloxane as a substitute for vitreous humour: characteristics and in vitro toxicity. J Funct Biomater. 2023;14(8):425. 37623669 10.3390/jfb14080425 PMC10455291

[21] Nusa HS, Astuti W, Kartasasmita AS, Virgana R, Syakir N, Bahtiar A, et al. Characterization of optical and structure properties of polydimethylsiloxanes. Mater Sci Forum. 2015;827:99–104.

[22] Chojnowski J, Cypryk M. Synthesis of linear polysiloxanes. In: Jones RG, Ando W, Chojnowski J, editors. Silicon-Containing Polymers: The Science and Technology of Their Synthesis and Applications. Dordrecht: Springer Netherlands; 2000. p. 3–41. 10.1007/978-94-011-3939-7_1.

[23] Mardina P, Prathama HA, Hayati DM. Pengaruh Waktu Hidrolisis Dan Konsentrasi Katalisator Asam Sulfat Terhadap Sintesis Furfural Dari Jerami Padi. Konversi. 2016;3(2):1.

[24] Septiani M, Auliya DG, Risdiana. Direct Comparison between Ring-Opening Polymerization and Hydrolysis-Condensation Methods in the Synthesis of Polydimethylsiloxane as a Vitreous Substitute: A Systematic Literature Review. Trends Sci. 2025. 10.48048/tis.2025.9668.

[25] Flory PJ. Fundamental principles of condensation polymerization. Chem Rev. 1946;39:137–97. 21000141 10.1021/cr60122a003

[26] Diana-Rivero R, Rivero DS, García-Martín A, Carrillo R, Tejedor D. Water as a reactant: DABCO-catalyzed hydration of activated alkynes for the synthesis of divinyl ethers. The Journal of Organic Chemistry. 2024;89(20):15068–74. 10.1021/acs.joc.4c01815. 39344303 10.1021/acs.joc.4c01815 PMC11494655

[27] Matsuoka I, Naito T, Yamada H. Relationship between the solubility of water in organic solvents and the extraction equilibrium. Bunseki Kagaku. 2002;51(9):759–65.

[28] Sediawan WB, Sulistyo H, Hidayat M. Sulfuric acid hydrolysis of various lignocellulosic materials and its mixture in ethanol production. Biofuels. 2009. 10.1080/17597269.2015.1110774.

[29] Twej W. Temperature Influence on The Gelation Process of Tetraethylorthosilicate Using Sol-Gel Technique. Iraqi J Sci. 2009;50:43–9.

[30] Villani V. Viscosity flow curves of agar and the bounded ripening growth model of the gelation onset. Molecules. 2024;29(6):1293. 38542931 10.3390/molecules29061293 PMC10975464

[31] Issa AA, Luyt AS. Kinetics of alkoxysilanes and organoalkoxysilanes polymerization: a review. Polymers. 2019. 10.3390/polym11030537. 30960521 10.3390/polym11030537 PMC6473841

[32] Djambo A. Effect of Temperature on The Efficiency of Polymerization Reactions Using Novel Catalysts in Cameroon. J Chem. 2024;3:36–47.

[33] Rutnakornpituk M. Synthesis of silicone magnetic fluids for use in eye surgery. Virginia Polytechnic Institute and State University. 2002.

[34] Soares JBP, Touloupidis V. Polymerization Kinetics and the Effect of Reactor Residence Time on Polymer Microstructure. In: Albunia AR, Prades F, Jeremic D, editors. Multimodal Polymers with Supported Catalysts: Design and Production. Cham: Springer International Publishing; 2019.

[35] Odian G. Principles of Polymerization. Chicago: John Wiley & Sons, Inc; 2004.

[36] Giordano G, Refojo MF. Silicone oils as vitreous substitutes. Prog Polym Sci. 1998;23:509–32.

[37] Rubinstein M, Colby RH. Polymer Physics. Oxford: Oxford University Press; 2003.

[38] Strobl G. The Physics of Polymers. Berlin: Springer Berlin Heidelberg; 2007.

[39] Callister WD, Rethwisch DG. Materials science and engineering: an introduction. Wiley; 2018.

[40] Saeedi F, Eshaghi A, Ramazani M, zabolian H, Esfahani AN. Transparent hydrophobic and anti-icing thin films based on polydimethylsiloxane-SiO2 nanoparticles co-modified with TMMS and HDMS. J Alloys Compd. 2025;1022:179921.

[41] Setiadji S, Sumiyanto E, Fitrilawati, Syakir N, Noviyanti AR, Rahayu I, et al. Synthesis of Polydimethylsiloxane and Its Monomer from Hydrolysis of Dichlorodimethylsilane. Key Eng Mater. 2020;860 KEM:234–8.

[42] Bai Y, Wang X, Wang X, Li Q, Yang K, Sun Y, et al. Study the intermolecular interaction of polymethylhydrosiloxane modifying acrylic resin and their application on anticorrosion area. Prog Org Coatings. 2024;195:108632.

[43] Cegłowski M, Schroeder G. Removal of heavy metal ions with the use of chelating polymers obtained by grafting pyridine–pyrazole ligands onto polymethylhydrosiloxane. Chem Eng J. 2015;259:885–93.

[44] Auliya D, Setiadji S, Fitrilawati F, Kartasasmita A, Risdiana R. Enhance the viscosity of polydimethylsiloxane by controlling the volume ratio of monomer and chain terminator. AIP Adv. 2024;14. 10.1063/5.0234700.

[45] Zahra NF, Auliya DG, Arini VF, Fitrilawati, Safriani L, Risdiana. NMR characterization of polymethylhydrosiloxane using dichloromethane and diethyl ether as solvent for vitreous substitute. ACS Omega. 2023;13(2):226–32.

[46] Silverstein RM, Webster FX, Kiemle DJ, Bryce DL. Spectrometric identification of organic compounds. Microchem J. 1963;7:388.

